# The Impact of a Culturally Adapted Patient Navigator-Led Telehealth Program for Displaced Afghans’ Mental Health

**DOI:** 10.1007/s10903-025-01758-y

**Published:** 2025-08-11

**Authors:** Qais Alemi, Hosai Todd Hesham, Jessica Cook, Wajma Naderi, Sadaf Afif

**Affiliations:** 1 Loma Linda University, Loma Linda, USA; 2 George Washington University, Washington D.C., USA; 3 Afghan Medical Professionals Association of America, Fairfax, VA USA; 4 Healis Health, Cambridge, MA USA; 5 Touro College of Osteopathic Medicine, New York, USA

**Keywords:** Afghan, Mental health, Patient navigator, Refugees, Telehealth

## Abstract

This study evaluated the effectiveness of a culturally- and linguistically-adapted, patient navigator-led telehealth model in expanding mental health access and reducing depression and anxiety symptoms among Afghan refugees resettled across several U.S. states. We conducted a retrospective chart review of electronic medical records from 679 Afghan patients, assessing changes in PHQ-9 and GAD-7 scores using paired samples t-tests. Additionally, we explored which patients benefited most, analyzing variations in outcomes based on treatment plans, age, and gender. We observed statistically significant reductions in depression and anxiety symptoms between baseline and follow-up for all treatment groups (*p* < .001), with large effect sizes ranging from d = 0.80 to 1.06. For example, high-need patients showed a mean PHQ-9 reduction of 4.44 points (d = 1.05) and a GAD-7 reduction of 3.70 points (d = 0.94). The model was equally effective across genders and age groups. This patient navigator-led model demonstrates a promising strategy for improving mental health outcomes among Afghan newcomers. Its success highlights the potential for scalable mental health care solutions to address both clinical symptoms and structural barriers to care.

## Background

Pioneered with cancer patients in the early 1990 s [1], *patient navigation* is designed to support patients in finding a way for integration and reduction of fragmentation, and to help them overcome barriers to accessing services [2]. Patient navigators are trained, culturally competent lay and professional healthcare workers, whose roles generally include streamlining appointments and paperwork for patients, helping patients arrive at scheduled appointments on time and prepared, and connecting them to appropriate social services [3, 4]. Prior studies indicate that patient navigator interventions can be effective in improving access to primary care, disease screening, and health outcomes for underserved groups in the United States (U.S.) including immigrants and refugees [5, 6].

Although patient navigation models hold promise in reducing health disparities, the effectiveness of navigator programs for refugees displaced by war, conflict, or persecution—who endure a disproportionately high burden of mental health challenges [7]—remains largely understudied. Despite the significant needs of refugees, accessing mental health care is an uphill battle and may remain so even years after resettlement due to difficulties navigating complex health systems in their countries of resettlement [8]. In addition, for Afghan refugees specifically, cultural and language barriers, lack of health insurance or covered services, lack of access to transportation, mistrust of service systems, mental health literacy, and stigma all stand in the way [9, 10].

In the present paper, we evaluate the effectiveness of a promising culturally- and linguistically-adapted patient navigator-led telehealth model for expanding mental health access and reducing depression and anxiety symptoms among Afghan refugees—dubbed the ***H****ealth* ***E****xtension for our* ***A****fghan A****L****lies* or the *HEAL Project*. In doing so, we focus on a recent wave of Afghans paroled in the U.S. via the federal government’s Operation Allies Welcome (OAW) program. Launched under the Biden Administration in August 2021 after the Taliban takeover, the initiative has aided over 76,000 vulnerable Afghans, including individuals working alongside U.S. military forces over the past two decades [11]. The program grants parolees temporary permission to lawfully reside in the U.S. for two years; however, it does not provide a dedicated path to permanent residency [12]. One avenue for seeking permanent residency includes applying for asylum. However, asylum approval is not guaranteed, leaving many Afghans in a state of uncertainty, fearing potential deportation amid an increasingly restrictive immigration climate [13].

Prior research has shown that uncertainties surrounding residency status, combined with a range of post-resettlement stressors, contribute to the mental health challenges faced by displaced Afghans [14, 15]. This underscores the importance of the present study, particularly in identifying effective treatments and interventions that may help alleviate mental health burdens and help Afghans heal. Here, we pursue the question of whether Afghan patients enrolled in the HEAL project experience clinically and statistically significant reductions in depression and anxiety symptoms between baseline and follow-up assessments. Additionally, we explore which patients benefit most from this patient navigator-led telehealth program by analyzing variations in support based on individual treatment plans, while also considering the influence of salient demographic characteristics such as age and gender. Findings from this study have the potential to inform similar efforts for future Afghan arrivals and other refugee populations utilizing patient navigator models.

## Methods

The study utilizes a single-group, pre-post retrospective chart review design to analyze electronic medical records from Afghan patients of all ages enrolled in the HEAL project between February 2023 and July 2024, as maintained by our partnered telemedicine platform. Our study protocol was reviewed and deemed exempt by Loma Linda University’s Institutional Review Board.

### Project Description

The HEAL project was implemented in January 2023 as one of the *Post-Resettlement Behavioral Health Support Program* projects by the U.S. Committee of Refugees and Immigrants. The project aims to expand mental health access and care for Afghan refugee adults, adolescents, and children through a telehealth platform. Utilizing the telehealth platform, Healis Health patients are connected with primary care physicians (PCPs) and/or psychiatrists with experience in mental health prescribing who are licensed across various U.S. states. The Afghan Medical Professional Association of America (AMPAA) appointed patient navigators to this project who were established Afghan refugees, many of whom were formerly medical providers in Afghanistan, fluent in Dari, Pashto, or Uzbek, and English. Their expertise spanned multiple specialties, including pediatrics, psychology, gynecology, otolaryngology, internal medicine, and public health.

To prepare for their roles, navigators received training in HIPAA patient privacy laws, mental health first aid, and life coaching, along with an introductory course on refugee mental health and resiliency. While navigators were not responsible for providing patient care, they played a central role in offering ongoing support throughout the referral and treatment process. Their responsibilities included addressing patient concerns, coordinating appointments, facilitating communication between patients and providers, and ensuring that patients received appropriate, culturally adapted, gender- and language-matched care. Given their shared lived experiences, navigators also provided peer support to foster rapport, reduce stigma, and enhance patient engagement.

### Procedures

This evaluation specifically examined the impact of the patient navigator component of the intervention, rather than the full clinical care package provided to patients (e.g., PCP visits, psychiatric medication, or therapy). While patients may have received additional services as part of their care, the current analysis is limited to understanding outcomes associated with their engagement with patient navigators. Patient data was initially analyzed based on the level of intervention required, categorized by mental health and psychosocial need. From this we created “treatment groups”, which included: (1) the “low-need” group who generally reported lower depressive and anxiety symptomatology and received support from both a patient navigator and a PCP; (2) the “moderate-need” group, which consisted of patients who, in addition to receiving support from a patient navigator and PCP, were also prescribed medication; and, (3) the “high-need” group, which included patients who required the same support as the moderate-need group plus individual or group therapy and/or access to local behavioral health support teams.

Throughout the project, patient navigators conducted an average of 26.6 weekly check-ins with patients, which included inquiries about their well-being, additional support needs, the effectiveness of prescribed medications, potential side effects, and any questions related to upcoming appointments. The overwhelming majority of patients joined the appointments through their smartphones. Some female and elderly patients had difficulty with *Zoom* and would join the appointment calls through *WhatsApp* instead. For a handful of patients who did not have secure access to the internet or smartphones, we coordinated with their case managers who assisted them in having them join their appointments from their office.

Data for this project were collected exclusively through standardized forms completed in the electronic medical record (EMR) during each navigator-patient interaction. Navigator episodes were not recorded, but session content was documented through these structured forms. The completed forms were extracted from the EMR and transferred to a HIPAA-compliant *Google Spreadsheet*, protected under a Business Associate Agreement. Although all patients signed standard clinical forms during enrollment—such as the Notice of Privacy Rights, Consent to Treatment, Client Release Authorization, and Telehealth Informed Consent—these forms did not include informed consent for research participation. As such, the HEAL Project’s Administrative Case Aide de-identified all data prior to sharing with the research team.

### Measurement and Data Analysis

The Patient Health Questionnaire-9 (PHQ-9) [16] and Generalized Anxiety Disorder-7 scale (GAD-7) [17] were administered to assess symptoms of depression and anxiety, respectively, both at baseline and follow-up. These measures were not validated for this group specifically; however, they have performed adequately in published studies testing psychological interventions with war-affected Afghans [18, 19]. These questionnaires were completed with the assistance of patient navigators prior to the patient’s initial appointment, which was then utilized by physicians to develop treatment plans. For the young children referred, their caretakers assisted and acted as the primary point of contact with the patient navigators. Caretakers would assist with form completions and also throughout the appointments.

Patient data for 679 available records from the EMR was transferred from the Google Spreadsheet and uploaded into SPSS (version 29.0) [20] for carrying-out all data analyses. We first screened for outliers and assessed the data for standard statistical assumptions, including skewness and kurtosis. Of the 679 participants who completed the baseline assessment, 149 were missing post-intervention (follow-up) outcome data for PHQ-9 and 143 for GAD-7. To assess the potential impact of missingness, we compared results from listwise deletion (complete-case analysis; *N* = 530 for PHQ-9 and *N* = 536 for GAD-7) with those obtained through regression imputation. Findings were nearly identical across both approaches, with no meaningful differences in means, test statistics, or p-values. Additionally, chi-square tests indicated that attrition at follow-up did not significantly differ by age, gender, residential area, or state of residence—both overall and within treatment groups—suggesting that data were likely missing at random. Given the minimal impact of missing data on our findings, the lack of significant attrition bias, listwise deletion was deemed appropriate for reporting primary results.

For descriptive purposes, we conducted univariate analyses to generate means, standard deviations, frequencies, and percentages where applicable. We also implemented independent samples t-tests and one-way analysis of variance (ANOVA) to examine relationships between our categorical descriptive variables along with PHQ-9 and GAD-7 scores. Moreover, we categorized the sample into three treatment groups: low-need, moderate-need, and high-need. We then performed paired samples t-tests to assess whether mean PHQ-9 and GAD-7 scores differed significantly between baseline and follow-up within each treatment group, applying a significance threshold of *p* <.05. Effect sizes (*Cohen’s d*) were calculated, where 0.2 represents a small effect, 0.5 a medium effect, and 0.8 a large effect [21]. Lastly, we implemented a series of analyses of covariance (ANCOVA) for each treatment group to examine whether follow-up PHQ-9 and GAD-7 scores varied by salient characteristics such as age and gender after controlling for baseline scores.

## Results

### Descriptive Analysis

Table 1 summarizes participant characteristics and baseline PHQ-9 and GAD-7 scores. In this non-random sample of 679 patients, young adults (20–34 years, 43.9%) were the largest group, while older adults (55+, 9.0%) were the smallest. Most were female, lived in suburban areas, and resided in Texas, Washington, Florida, or Pennsylvania. The majority were in the high-need treatment group, followed by moderate- and low-need groups. ANOVA results showed significant overall models for age and PHQ-9/GAD-7 scores, though pairwise PHQ-9 comparisons were nonsignificant due to high within-group variability. Though for GAD-7, older adults reported significantly higher anxiety than the child and adolescent group. T-tests found no gender differences in PHQ-9, but females had significantly higher GAD-7 scores. Residential area was unrelated to PHQ-9/GAD-7, but states with behavioral health teams had lower depression and anxiety levels. Both PHQ-9 and GAD-7 scores varied significantly by treatment group, with higher scores in higher-need group. Pairwise comparisons were significant for PHQ-9 (low vs. high, moderate vs. high) and for all GAD-7 treatment group comparisons.

**Table 1:** Characteristics of study participants (*N* = 679)

Variable	Category	*n* (%)	Baseline PHQ-9 Mean (SD)	Baseline GAD-7 Mean (SD)
Age Categories	4–19 (Children & Adolescents)	86 (12.6)	6.08 (4.94)	4.93 (3.86)
	20–34 (Young Adults)	298 (43.9)	7.21 (4.94)	5.92 (4.08)
	35–54 (Middle-Aged Adults)	234 (34.5)	6.36 (4.43)	5.53 (4.11)
	55+ (Older Adults)	61 (9.0)	7.93 (5.38)	6.98 (4.74)
p-value			0.026	0.019
Gender	Male	297 (43.7)	7.27 (4.62)	6.04 (3.92)
	Female	382 (56.3)	7.64 (3.92)	6.70 (3.84)
p-value			0.340	0.010
Residential Area^b^	Rural	49 (7.2)	6.71 (4.13)	5.59 (3.97)
	Suburban	343 (50.5)	6.99 (4.96)	5.82 4.07)
	Urban	285 (42.0)	6.69 (4.82)	5.70 (4.29)
p-value			0.776	0.875
State of Residence	^a^TX, WA, FL, PA	355 (52.3)	6.19 (4.51)	5.19 (3.91)
	Other States + District of Columbia	324 (47.7)	7.55 (5.08)	6.36 (4.31)
p-value			< 0.001	< 0.001
Treatment Groups	Low-Need	171 (25.2)	5.62 (4.64)	4.42 (3.92)
	Moderate-Need	213 (31.4)	6.64 (4.02)	5.58 (3.69)
	High-Need	295 (43.4)	7.69 (5.31)	6.66 (4.32)
p-value			< 0.001	< 0.001

^a^U.S. states with community behavioral health field teams

^b^Two missing cases

### Inferential Analysis

Table 2 presents results of the paired t-tests comparing baseline and follow-up scores for the PHQ-9 and GAD-7 across the three treatment groups: low-, moderate-, and high-need. In every treatment group, PHQ-9 and GAD-7 scores significantly decreased from the initial assessment to the follow-up, and all of the changes in mean scores were statistically significant (*p* <.001). Initial PHQ-9 and GAD-7 scores were higher in the moderate- and high-need groups, suggesting greater symptom severity at baseline. These groups also experience the larger absolute reductions in scores. Moreover, the Cohen’s d values suggest large effect sizes for most comparisons, indicating clinically meaningful reductions in both depression and anxiety symptoms. The lowest effect size is 0.80 for anxiety in the low-need group, and the highest effect size is 1.06 for both depression and anxiety in the moderate-need group.

**Table 2:** Paired sample t-tests for PHQ-9 and GAD-7 scores across treatment groups

Treatment group	Measure	Follow-up score M (SD)	Mean score difference (95% CI)	Cohen’s d	t-statistic (df), *p*-value
Low-Need	PHQ-9	1.77 (2.13)	3.27 (2.53, 4.00)	0.87	9.45 (116), < 0.001
	GAD-7	1.45 (1.97)	2.42 (1.87, 2.96)	0.80	8.637 (117), < 0.001
Moderate-Need	PHQ-9	2.51 (2.76)	3.67 (3.08, 4.25)	1.06	13.37 (157), < 0.001
	GAD-7	2.15 (2.52)	3.23 (2.73, 3.74)	1.06	13.91 (160), < 0.001
High-Need	PHQ-9	3.08 (3.25)	4.44 (3.86, 5.02)	1.05	16.71 (254), < 0.001
	GAD-7	2.87 (3.13)	3.70 (3.19, 4.21)	0.94	14.32 (256), < 0.001

### Multivariate Analysis

We conducted ANCOVAs to examine whether follow-up PHQ-9 and GAD-7 scores varied by age and gender (not shown). After controlling for baseline depression and anxiety measures (along with state of residence, which indicates availability of local teams behavioral health support), ANCOVAs indicated that the overall models predicting follow-up PHQ-9 scores were statistically significant for the low-, moderate-, and high-need groups (*p* <.001). However, neither age category (*p* =.286, 0.273, 0.396, respectively) nor gender (*p* =.423, 0.243, 0.549, respectively) significantly predicted follow-up PHQ-9 scores. In contrast, initial PHQ-9 scores were strong and significant predictors across all groups (*p* <.001). Similarly, for GAD-7 scores, the overall models were statistically significant for the low-, moderate-, and high-need groups (*p* <.001). In the low-need group, age was significantly associated with GAD-7 scores (*p* =.006). In the moderate-need group, neither age (*p* =.813) nor gender (*p* =.449) significantly predicted follow-up GAD-7 scores. Similarly, in the high-need group, neither age (*p* =.893) nor gender (*p* =.106) were significant predictors of follow-up GAD-7 scores. Across all groups, initial GAD-7 scores were strong and significant predictors (*p* <.001). In sum, these findings suggest that the interventions were equally effective across different age groups and for both males and females in terms of reducing depressive symptoms. However, findings indicate that younger patients may have benefited more in terms of reducing anxiety symptoms.

## Discussion

The purpose of this study was to evaluate the effectiveness of a patient navigator-led telehealth program in reducing depression and anxiety symptoms among recently resettled Afghans paroled across multiple U.S. states. This population is distinct from prior studies of resettled Afghans in the U.S. due to the lack of assurance regarding their long-term legal status and the possibility of repatriation to Afghanistan. This prolonged legal limbo contributes to significant psychological distress [14, 15], compounding the trauma of displacement and increasing vulnerability to depression and anxiety.

We conducted a retrospective analysis of electronic medical records from patients enrolled in the HEAL project, a program that, in collaboration with its partnered telehealth platform, provided mental health support and services to Afghan parolees, which was facilitated by patient navigators. Our results show significant reductions in depression and anxiety symptoms across all treatment groups. In addition, the large effect sizes underscore the clinical significance of these improvements, demonstrating substantial mental health benefits for participants, whether they received support solely from a patient navigator or in combination with medication, therapy, and/or psychosocial support. Moreover, our regression analysis provides some indication that younger versus older patients in the low-need group may have benefited more from the support offered by patient navigators. Additionally, our data also suggests that the intervention was equally beneficial for males and females regardless of treatment group.

We argue that the success of this patient navigator-led program is largely attributable to its cultural and linguistic adaptations, which were designed to address the unique barriers Afghan refugees often face when seeking care, particularly stigma. By offering peer support, patient navigators may have helped normalize professional help-seeking, fostering a safe space where Afghan refugees could manage their mental health without fear of judgment. Additionally, delivering services through telehealth and PCPs rather than potentially stigmatizing mental health clinics [22] may have further reduced barriers to care. While telehealth service delivery for refugees presents challenges—including structural and technological barriers as well as varying levels of patient digital literacy [23]—patient navigators played a crucial role in mitigating these obstacles here.

Since the literature on patient navigator models for refugees is limited, direct comparisons between our findings and previous studies are not feasible. Though our findings align with the broader literature on similar community health worker (CHW)-delivered interventions for reducing mental health challenges in underserved populations [24]. Like many CHW models, we employed navigators that for the most part began with no formal mental health training. A unique aspect of our approach was the appointment of patient navigators who were established refugees and formerly medical professionals in various fields in Afghanistan. While they were not licensed to practice in their respective fields in the U.S., their expertise and role as trusted figures may have been both impactful for patients and empowering for themselves.

Future qualitative research is needed to explore this dual impact, including the potential for countertransference, and the emotional support needs of patient navigators as they assist individuals experiencing severe trauma and mental health challenges. More research is also needed to confirm the positive impact of our patient navigator-led model that was specific to facilitating professional mental health care for Afghan refugees. Future research focusing on the expanded role of navigators in mitigating post-resettlement stressors that often exacerbate pre-migration traumas and poor mental health will also be necessary. In addition, longitudinal research is essential to determine the long-term effectiveness of patient navigator models for Afghans and other displaced populations. However, the implementation of more rigorous research designs, including controlled experiments should be considered against an array of feasibility and acceptability challenges observed in refugee communities [25].

In relation to this, the absence of a control group is the most critical limitation of our study, which prevents us from establishing causality between the intervention and the observed changes in PHQ-9 and GAD-7 scores. Another limitation here is the use of the GAD-7 and PHQ-9, which are not validated specifically for Afghans and may have led to inaccuracies in the measurement of symptoms. Culturally grounded measures, such as the Afghan Symptom Checklist [26], which captures idioms of distress familiar to Afghans, may have provided a more precise assessment of symptomatology. Also, we did not collect data on educational attainment, employment or other social factors that could have mediated the relationships observed here. Nonetheless, our findings align with the broader literature demonstrating that culturally competent guidance provided by navigators from patients’ own ethnic communities help overcome barriers to care [5]. This carries important policy implications that call for integrating patient navigation models into primary care, behavioral health, and community health settings, along with investing in training programs for both lay and professional navigators from refugee communities.

### New Contribution to the Literature

In this study we show that the HEAL project, a culturally- and linguistically-adapted patient navigator-led telehealth model is promising in reducing depression and anxiety symptoms among displaced Afghans recently resettled across several U.S. states. As far as we know this is the first study that examines the role of patient navigators in assisting refugees in terms of accessing and utilizing professional health services to improve their mental health. Hence study also contributes to the scant evidence on the use of telehealth models with refugees. Its success in the Afghan parolee population suggests potential applicability to other displaced groups facing similar challenges.

## Data Availability

The data that support the findings of this study are derived from de-identified patient records and contain sensitive information. Due to privacy and ethical restrictions, the dataset is not available for public access.
